# Co-delivery of doxorubicin and curcumin by pH-sensitive prodrug nanoparticle for combination therapy of cancer

**DOI:** 10.1038/srep21225

**Published:** 2016-02-15

**Authors:** Yumin Zhang, Cuihong Yang, Weiwei Wang, Jinjian Liu, Qiang Liu, Fan Huang, Liping Chu, Honglin Gao, Chen Li, Deling Kong, Qian Liu, Jianfeng Liu

**Affiliations:** 1 Tianjin Key Laboratory of Radiation Medicine and Molecular Nuclear Medicine, Institute of Radiation Medicine, Chinese Academy of Medical Science & Peking Union Medical College. Tianjin 300192, P.R. China; 2 Tianjin Key Laboratory of Biomaterial Research, Institute of Biomedical Engineering, Chinese Academy of Medical Science and Peking Union Medical College, Tianjin 300192, P.R. China; 3 Department of Urology, Tianjin First Central Hospital, Tianjin 300192, P. R. China

## Abstract

Ample attention has focused on cancer drug delivery via prodrug nanoparticles due to their high drug loading property and comparatively lower side effects. In this study, we designed a PEG-DOX-Cur prodrug nanoparticle for simultaneous delivery of doxorubicin (DOX) and curcumin (Cur) as a combination therapy to treat cancer. DOX was conjugated to PEG by Schiff’s base reaction. The obtained prodrug conjugate could self-assemble in water at pH 7.4 into nanoparticles (PEG-DOX NPs) and encapsulate Cur into the core through hydrophobic interaction (PEG-DOX-Cur NPs). When the PEG-DOX-Cur NPs are internalized by tumor cells, the Schiff’s base linker between PEG and DOX would break in the acidic environment that is often observed in tumors, causing disassembling of the PEG-DOX-Cur NPs and releasing both DOX and Cur into the nuclei and cytoplasma of the tumor cells, respectively. Compared with free DOX, free Cur, free DOX-Cur combination, or PEG-DOX NPs, PEG-DOX-Cur NPs exhibited higher anti-tumor activity *in vitro*. In addition, the PEG-DOX-Cur NPs also showed prolonged blood circulation time, elevated local drug accumulation and increased tumor penetration. Enhanced anti-tumor activity was also observed from the PEG-DOX-Cur-treated animals, demonstrating better tumor inhibitory property of the NPs. Thus, the PEG-DOX-Cur prodrug nanoparticle system provides a simple yet efficient approach of drug delivery for chemotherapy.

There are several limitations concerning systemic administration of single-drug chemotherapy, including fast blood/renal clearance, poor bioavailability and multidrug resistance. In addition, drug accumulation at the tumor sites is often too low to reach an effective dose, requiring high drug dose during administration which in turn causes severe adverse side effects^1^. Besides, single drug chemotherapies may not be potent enough to suppress all cancer cell growth given the inhomogeneous distribution of cancer cells within the tumors^2^. Recently, combination chemotherapy of multiple anticancer drugs has been extensively developed since it could reduce multidrug resistance and side effects as a result of lower dosage of each drug^3^. However, it remains challenging to obtain significant antitumor effect with reduced normal tissue toxicity since different drugs possess different physical and chemical properties^4^,^5^.

Nanocarriers, such as hydrogels^6^,^7^,^8^,^9^,^10^,^11^, polymeric nanoparticles^12^,^13^,^14^,^15^, liposomes^16^,^17^,^18^, and self-assembling nanofibers^19^,^20^ have all been reported to enhance the therapeutic efficiency of anticancer drugs by facilitating local drug accumulation, developing drug bioavailability, and prolonging systemic circulation^21^. More importantly, they also provide a possible approach by incorporating two or more drugs into one nanocarrier. Due to the passive targeting ability by the enhanced permeability and retention (EPR) effect^22^, nanocarriers could preferentially deliver the loaded drugs into tumor tissues and improve anti-tumor efficacy^23^,^24^. However, despite the unique advantages of nanotechnology in cancer therapy, some nanocarriers with poor loading ability require multiple injections to achieve ideal therapeutic effect, which could result in systemic toxicity and serious inflammatory response^25^. Meanwhile, the “low drug loading” nanocarrier could not completely overcome multidrug resistance caused by exposure to free drugs. Hence, it is highly desirable to develop a combination drug delivery system with high drug load and multi-drug delivery property.

The stimuli-responsive prodrug nanoparticles represent a new generation of intelligent and effective drug delivery systems. Generally, the small drug molecule is directly conjugated to a (macro)molecule, which remains as an inactive prodrug in blood circulation and only turns active at the targeted sites^26^,^27^. In addition, the prodrug nanoparticle systems have also been shown to improve drug loading efficiency and reduce nanocarrier dosage compared to the conventional nanosized vehicles.

In the present study, we adopted a “conjugation to” strategy^28^,^29^,^30^,^31^,^32^ and designed, synthesized, and evaluated a simple yet efficient anti-tumor prodrug nanoparticle as cancer drug delivery system. The prodrug nanoparticle (Fig. 1A) was composed of a methoxy-poly (ethylene glycol)-aldehyde (mPEG-CHO) chain and an anthracycline antibiotic, doxorubicin (DOX). The amino group of DOX could react with the terminal aldehyde group of PEG by Schiff’s base reaction to form PEG-DOX, which was capable of self-assembling into pH-sensitive prodrug nanoparticle (PEG-DOX NPs) in aqueous environment at pH 7.4. Given the hydrophobic nature of curcumin (Cur), a natural polyphenol, could be entrapped within the PEG-DOX NPs through hydrophobic interactions, resulting in a prodrug nanoparticle (PEG-DOX-Cur NPs) system for co-delivery of both DOX and Cur (Fig. 1B). The size of the PEG-DOX-Cur NPs is suitable to avoid rapid blood clearance, enable passive targeting through EPR effect (Fig. 1C) and enhance cellular internalization (Fig. 1D). Moreover, under the acidic environment that is often found in tumors, the Schiff’s base groups within the nanoparticles would break and release both anti-cancer drugs at tumor sites (Fig. 1E). Our *in vitro* and *in vivo* anti-tumor studies revealed better inhibitory effect on tumor growth in the PEG-DOX-Cur NPs-treated animals than those treated by free DOX/Cur mixture. Drug accumulation and penetration studies also indicated that the PEG-DOX-Cur NPs possessed prolonged blood circulation time, better local drug accumulation and enhanced drug penetration in tumors, further demonstrating a superior effect of the PEG-DOX-Cur NPs in cancer drug delivery.

## Results

### Synthesis and characterization of PEG-DOX NPs

**Figure 2:**
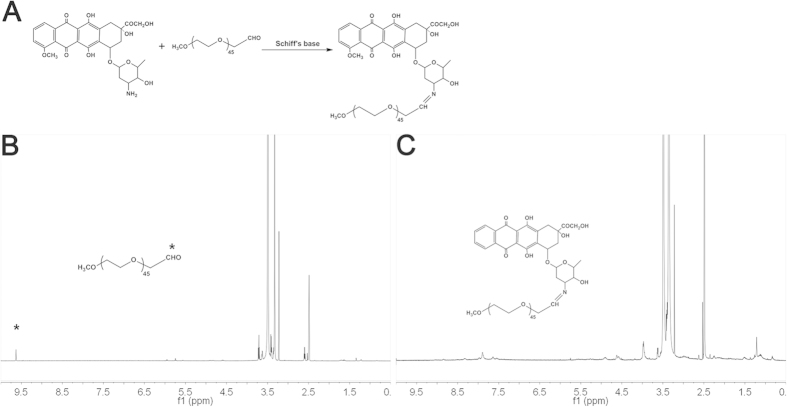
The synthetic route to PEG-DOX (**A**) and ^1^H NMR spectra of mPEG-CHO (**B**) and PEG-DOX (**C**).

**Figure 3:**
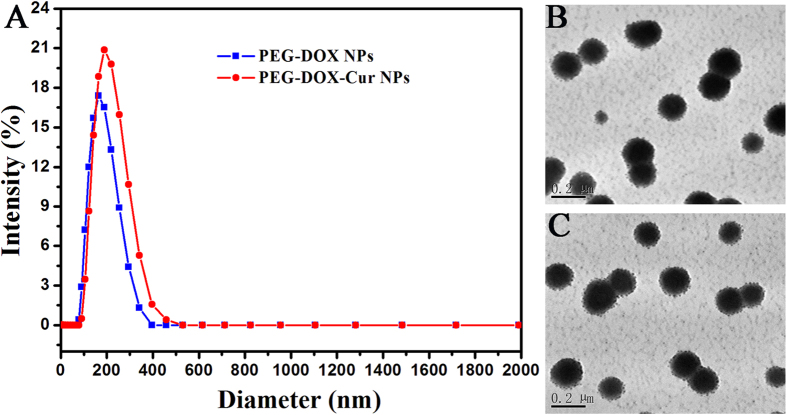
Hydrodynamic size distributions (**A**) and TEM images of PEG-DOX (**B**) and PEG-DOX-Cur NPs (**C**).

PEG-DOX was synthesized by the Schiff’s base reaction between the end aldehyde group of mPEG (M_n_ = 2000) and the amine group of DOX molecule (Fig. 2A). The successful conjugation was confirmed by the disappearance of characteristic chemical shift at 9.63 ppm assigned to the hydrogen proton of aldehyde group (-CHO) and the appearance of characteristic peaks attributed to DOX in the ^1^H NMR spectrum (Fig. 2B,C). Cur was then encapsulated into the PEG-DOX NPs by the hydrophobic interaction, achieving the simultaneous loading of DOX and Cur in one nanocarrier (PEG-DOX-Cur NP). The drug loading content was measured by UV-vis and the hydrodynamic diameter as well as the morphology of nanoparticles was also determined by DLS and TEM, respectively. As summarized in Table 1 and Fig. 3, the PEG-DOX-Cur NPs showed a weak negative charged surface and an average hydrodynamic diameter of around 183.5 nm, which was slightly larger than that of PEG-DOX NPs (161.8 nm). Under TEM observation, both PEG-DOX and PEG-DOX-Cur NPs exhibited a spherical morphology. The drug loading content of DOX and Cur was determined to be as high as 18.35% and 18.2%, respectively. Compared with the traditional structure of nanocarrier^33^,^34^, our designed PEG-DOX-Cur NPs possessed higher drug loading capacity, as the drugs directly acted as the hydrophobic core. The result suggested that the prodrug nanoparticle can co-deliver multiple therapeutic agents with a single nanocarrier.

### *In vitro* acid-triggered drug release study

**Figure 4:**
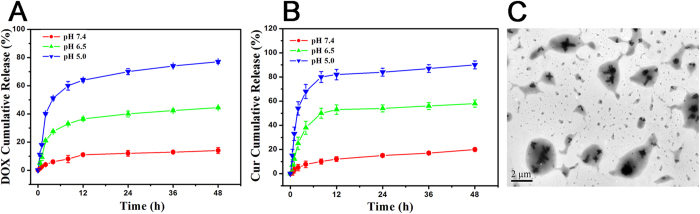
Drug release profiles of PEG-DOX-Cur NPs, (**A**) DOX of PEG-DOX-Cur NPs and (**B**) Cur of PEG-DOX-Cur NPs) under different pH values. Data were expressed as mean ± SDs (n = 3). (**C**) The morphology of PEG-DOX-Cur NPs after incubation at pH 5.0 for 48 h.

The oxime bond formed by Schiff’s base between PEG and DOX could specifically be hydrolyzed under acid environments^35^,^36^, which would result in the disassembly of nanoparticles and the acceleration of the drug release. As depicted in Fig. 4A, DOX was quickly released from PEG-DOX-Cur NPs at pH 5.0 within 24 h due to the breakage of oxime bond. The Cur encapsulated by PEG-DOX-Cur NPs displayed a similar release profile at the acid condition, as shown in Fig. 4B. This might be ascribed to the disassembly of nanoparticles at the acid environment. Meanwhile, as shown in Fig. 4C, the morphology of the nanoparticle was disintegrated and irregularly aggregated under acid condition for 48 h. The results revealed that our designed prodrug nanoparticle provided a platform to simultaneously release multiple therapeutic agents under acid condition of tumor microenvironment.

### Cellular uptake and intracellular drug localization

**Figure 5:**
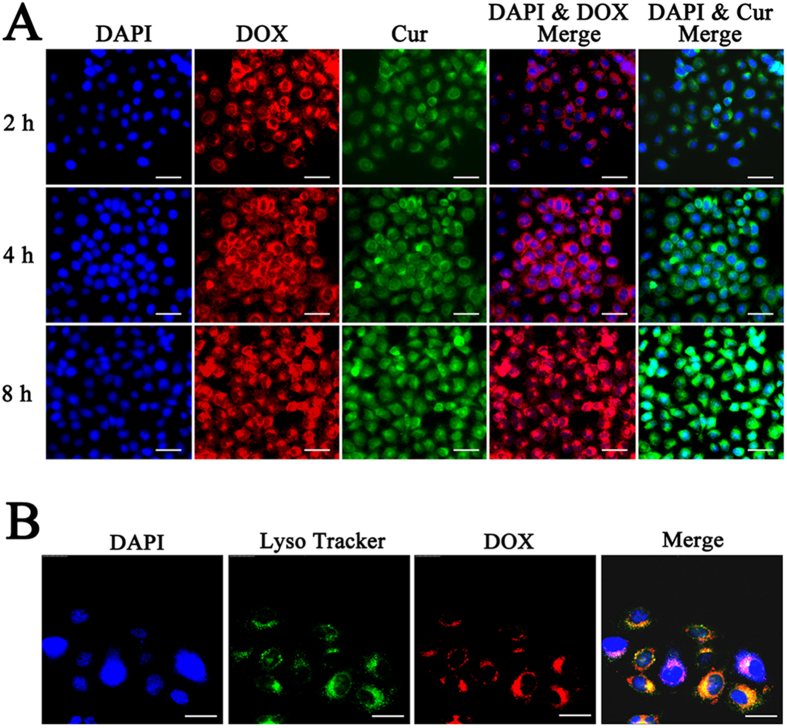
Cellular uptake and localization of PEG-DOX-Cur NPs in HepG 2 cancer cells observed by fluorescence microscopy after incubation for 2, 4, and 8 h, respectively. Scale bar = 40 μm (**A**). Colocalization studies of PEG-DOX-Cur NPs carried out using lysosome tracker in HepG 2 cells after incubation for 0.5 h. Scale bar = 25 μm (**B**).

The cellular uptake and internalization of PEG-DOX-Cur NPs were investigated using HepG 2 cancer cells by fluorescence microscopy based on the intrinsic red fluorescence of DOX and green fluorescence of Cur. DAPI with blue fluorescence was employed for labeling the cell nucleus as well. As shown in Fig. 5A, PEG-DOX-Cur NPs could be efficiently taken up by HepG 2 cells, which was predominately distributed in cytoplasm after 2 h of incubation. Fluorescence images acquired after incubation for 4 h displayed high fluorescence intensity of drugs (DOX and Cur) within cells, and after 8 h of incubation, the fluorescence intensity of DOX in the cell nucleus was significantly stronger than that in cytoplasm. As free DOX is mainly distributed in cell nucleus and exert drug effects through intercalation with DNA and inhibition of macromolecular biosynthesis^37^,^38^, this result indicated that the DOX had been released from PEG-DOX-Cur NPs. On the other hand, the fluorescence intensity of Cur in the cytoplasm increased as the time elapsed. As Cur can increase the levels of topoisomerase II-mediated DNA cleavage in the cytoplasm through its natural antioxidant activity^39^, the increased concentration of Cur in the cytoplasm may bring enhanced anticancer effects. These results indicated that PEG-DOX-Cur NPs could be efficiently uptaken by cancer cells, ensuring the consistent intracellular release of DOX and Cur. The cellular location of PEG-DOX-Cur NPs was shown in Fig. 5B, the DOX signals colocalized well with lysosome tracker, where the yellow regions highlighted the colocalized areas. This result indicated that PEG-DOX-Cur NPs were uptaken by cells *via* endocytosis.

**Figure 6:**
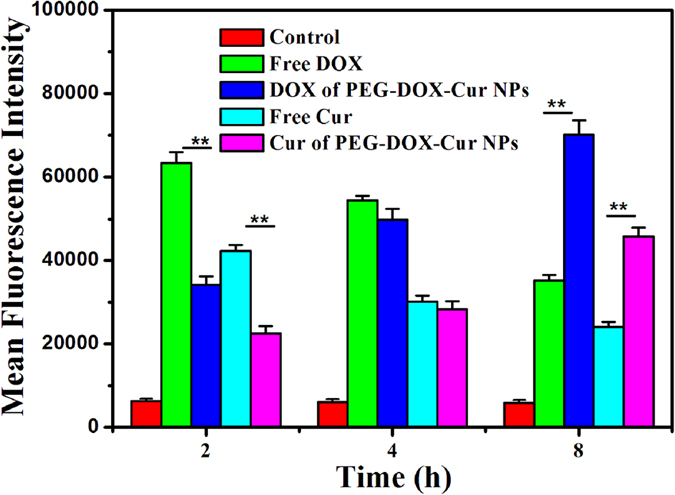
Quantitative analysis of mean fluorescence intensity after incubated with PEG-DOX-Cur NPs and free drugs for 2, 4, and 8 h through flow cytometry. (Blank cells as the control, **P < 0.01 in comparison with DOX and Cur, respectively).

The cellular uptake of PEG-DOX-Cur NPs was also quantitatively determined by flow cytometry. As shown in Fig. 6, both free DOX and free Cur possessed highest fluorescence intensity after the cells were incubated with each drug for 2 h, which significantly decreased with the incubation time elapsed. On the contrary, after the HepG 2 cells were incubated with the PEG-DOX-Cur NPs for 2 h, the fluorescence intensity of both DOX and Cur was lower than free DOX or Cur incubation at 2 h time point. Noteworthy is that the fluorescence intensity of DOX and Cur greatly enhanced when the treatment time of the HepG 2 cells with nanoparticles increased. After 8 h incubation, the fluorescence intensity of DOX and Cur from PEG-DOX-Cur NPs group was significantly higher than the corresponding free drug groups (P < 0.01). It is well-known that free small molecule drugs can quickly penetrate cellular and nuclear membranes *via* passive diffusion, whereas the drug-loaded nanoparticle is taken up by the endocytosis pathway^35^,^40^,^41^. The different cell uptake profiles of PEG-DOX-Cur NPs and free drugs should be attributed to the above-mentioned different cellular uptake pathways.

### *In vitro* cytotoxicity studies

**Figure 7:**
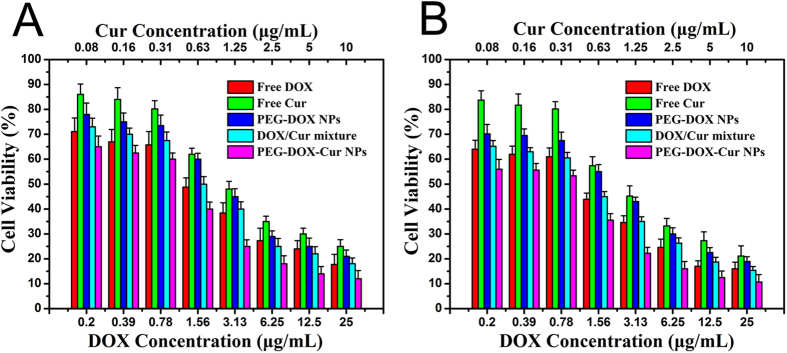
Cytotoxicity of free drugs, PEG-DOX NPs, PEG-DOX-Cur NPs and free drug mixtures against HepG 2 (**A**) and HeLa (**B**) cancer cells. Data were expressed as mean ± SDs (n = 6).

In order to verify the enhanced anticancer effect of the PEG-DOX-Cur NPs, the proliferation inhibition of PEG-DOX-Cur NPs was tested against HeLa and HepG 2 cancer cells. Free DOX, free Cur, DOX/Cur mixture as well as PEG-DOX NPs were used as the controls. As shown in Fig. 7, the cell proliferation inhibition efficacies of all the samples exhibited a strongly dose-dependent pattern after culture for 24 h. Although the cell suppression capacity of PEG-DOX NPs was weaker than free DOX, the NPs still showed effective anti-tumor activity *in vitro*. It is encouraging that the anti-tumor activity of PEG-DOX-Cur NPs was obviously higher than the other four groups, especially in the range of higher drug concentration. The IC_50_ values of the samples were evaluated in Table 2. The IC_50_ value of DOX/Cur mixture was similar to DOX, indicating that the simple combination of free Cur and DOX did not achieve obvious enhancement effect. This result may be caused by quick internalization and removal of free DOX and Cur through passive diffusion by cancer cells, as shown in Fig. 7, resulting in a short anti-tumor time. The IC_50_ value of PEG-DOX-Cur NPs was much lower than free DOX and DOX/Cur mixture. This should be because the PEG-DOX-Cur NPs were disassembled after internalized by cancer cells, and meanwhile, the DOX and Cur released by PEG-DOX-Cur NPs were continuously accumulated within the tumor cells, and then played a role in killing cancer cells. The *in vitro* cytotoxicity results indicated that DOX and Cur released from PEG-DOX-Cur NPs could play enhanced anti-tumor effect.

### *In vivo* drug distribution and tumor accumulation studies

**Figure 8:**
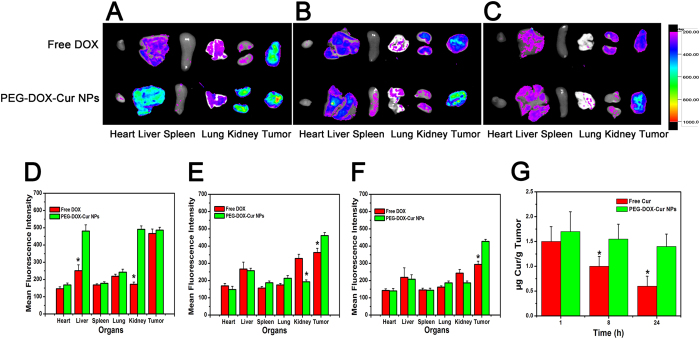
Representative *ex vivo* images and mean fluorescence intensity of tumor and main organs (heart, liver, spleen, lung and kidney). Tissues were excised at 1 (**A,D**), 8 (**B,E**) and 24 h (**C,F**) after administration of DOX and PEG-DOX-Cur NPs and subjected to the imaging equipment. Quantitative analysis the concentration of Cur in tumor tissues after administration of Cur and PEG-DOX-Cur NPs (**G**). (*P < 0.05 in comparison with DOX or Cur).

The *in vivo* DOX distribution and tumor accumulation of free DOX and PEG-DOX-Cur NPs in HepG 2 tumor-bearing nude mice were assessed by taking advantage of the DOX fluorescence. After intravenous injection of DOX or PEG-DOX-Cur NPs, respectively, the mice were sacrificed at 1, 8 and 24 h post-injection, respectively, and then the major organs as well as tumors were collected and imaged. As shown in Fig. 8A–F, for free DOX group, the DOX fluorescence was mainly distributed in tumor at 1 h post-injection. The low fluorescence intensity in the main organs indicated that free DOX could be excreted quickly from the body. This result verified the short half-life and rapid clearance of DOX^42^,^43^,^44^. It is also noted that the fluorescence intensity in tumor for free DOX group was greatly reduced after injection for 8 h and 24 h, respectively, indicating the low retention of free DOX in tumor. On the other hand, the fluorescence signals of PEG-DOX-Cur NP-treated group were mainly localized in the liver and kidney and highly accumulated in the tumor at 1 h post-injection. At 24 h post-injection, the DOX fluorescence intensities in the main organs became very weak for PEG-DOX-Cur NPs groups. In comparison, the fluorescence intensity in tumor tissues of PEG-DOX-Cur NP-treated mice was still strong. This result was mainly attributed to the tumor acid environment-triggered DOX release feature of PEG-DOX-Cur NPs, which avoided significant drug leakage in the blood circulation.

The weakly fluorescence intensity of Cur and poor specificity resulted in unsatisfactory distribution data by *in vivo* imaging. It obliged us to detect the concentrations of Cur in the tumors by LC-MS/MS. The mainly data was shown in Fig. 8G, the variation tendency of Cur accumulated in tumors was similar to DOX. The similar results of drugs accumulating in tumors further verified the consistency of tumor accumulations between DOX and Cur, which mainly attributed to the unique release characteristics of PEG-DOX-Cur NPs.

**Figure 9:**
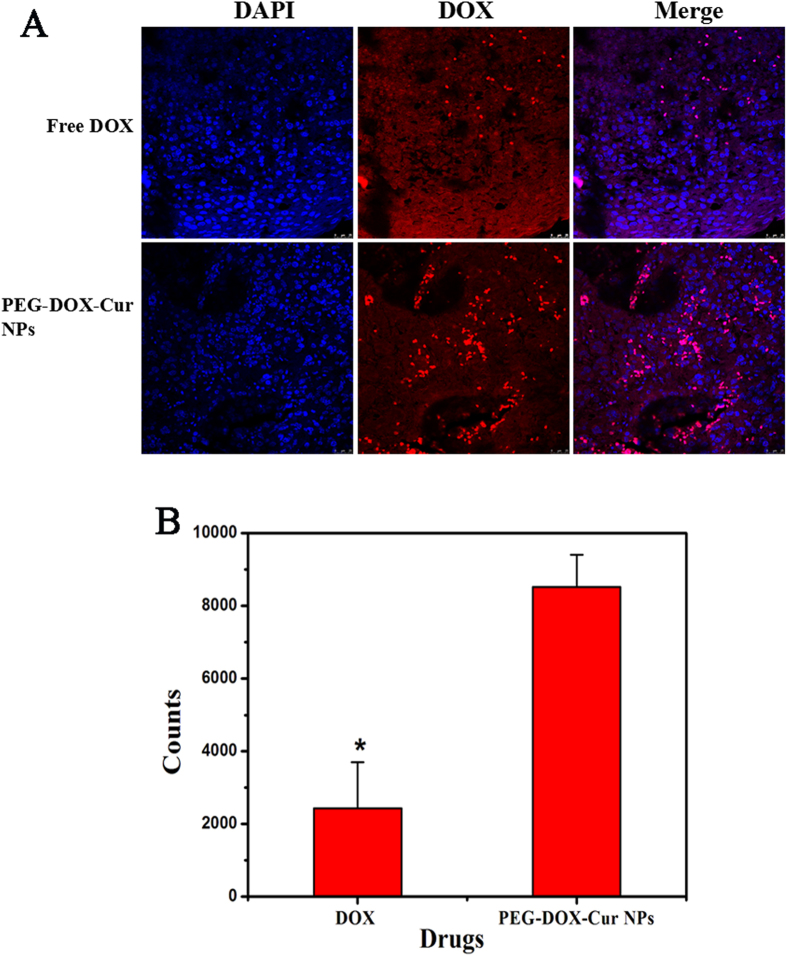
Rough measurement of tumor penetrating efficiency of PEG-DOX-Cur NPs and DOX (**A**) and the statistical data (**B**) of red signals in tumor sections. Tumors were removed at 24 h after different formulations administration. (*p < 0.05 in comparison with DOX).

Subsequently, the accumulation and penetration of DOX in tumors were roughly assessed by the tumor sections from both free DOX-treated and PEG-DOX-Cur NPs treated mice. The fluorescent images and the fluorescence intensity statistics were shown in Fig. 9. Compared to free DOX treatment, prodrug nanoparticles showed enhanced accumulation of DOX in tumor as well as more extensive distribution within tumor, which was consistent with the result of excised tumor imaging.

### *In vivo* anti-tumor activity

**Figure 10:**
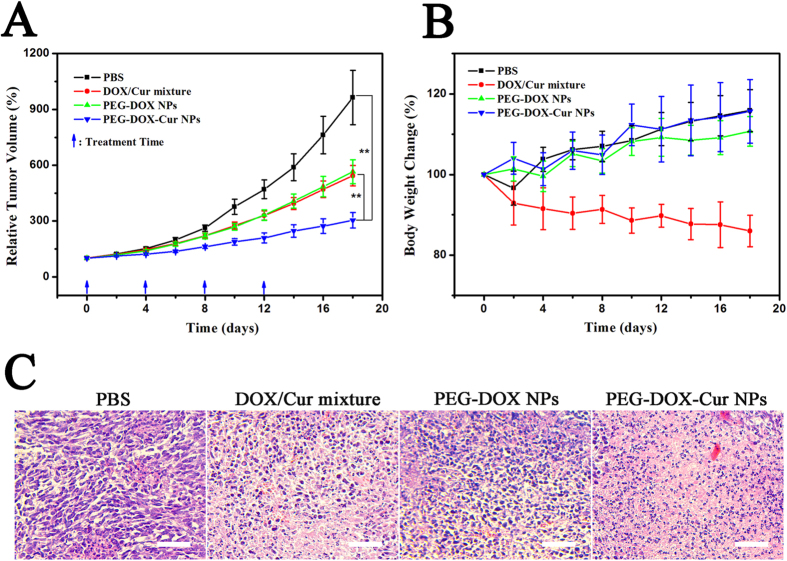
*In vivo* anti-tumor effects of DOX/Cur mixture, PEG-DOX NPs and PEG-DOX-Cur NPs on BALB/c nude mice (male, 6–8 weeks) bearing HepG2 xenografts. (**A**) Relative tumor volume of different treatment groups (n = 10); (*p < 0.05, **p < 0.01). (**B**) Relative body weight change after treatment. Data expressed as mean ± standard deviation values. (**C**) H&E assays of tumors from HepG2 tumor xenograft-bearing BALB/c nude mice after being received different treatments for 18 days. Scale bar = 50 μm.

The anti-tumor results of different formulations were shown in Fig. 10A, the tumor growth trend of different treatment groups was much slower than PBS group. Compared with the tumor volume treated with DOX/Cur mixture, the tumor volume treated with PEG-DOX NPs had no statistical differences, which was consistent with the *in vitro* cytotoxicity results. We should select multiple doses to evaluate the anti-tumor effects between PEG-DOX NPs and DOX/Cur mixture.

The tumor volume treated with PEG-DOX-Cur NPs was significantly smaller than the tumor volume treated with DOX/Cur mixture and PEG-DOX NPs. We can summarized that the combination anti-tumor effects from PEG-DOX-Cur NPs was better than the free drugs mixture, which attributed to the consistency of release kinetics by the two loaded drugs. DOX and Cur could be delivered into tumor cells simultaneously by the EPR effect, and then combine killing cancer cells after being released by PEG-DOX-Cur NPs. The superior anti-tumor effect of PEG-DOX-Cur NPs might be also ascribed to the sufficient cellular uptake in the tumors and the enhanced effect of DOX and Cur on tumor inhibition.

The body weight indicators were monitored during the treatment. As shown in Fig. 10B, the variation tendency of different groups was obviously different, thereinto, the mice treated with DOX/Cur mixture showed obviously loss weight, demonstrating that side effects were produced by DOX or DOX/Cur mixture. However, the body weight of PEG-DOX-Cur NPs and PEG-DOX NPs treated mice showed a slow and sustained increase, which may be attributed to the reduced toxicity of the loaded drugs.

To further investigate the anti-tumor efficacy of different treatment formulations, the BALB/c nude mice bearing HepG2 tumor model of different groups were randomly sacrificed after treatment, tumor sections were prepared and stained with hematoxylin and eosin (H&E) for pathology analysis. The pathological results were shown in Fig. 10C, all treatment groups showed various levels of necrosis, the PEG-DOX-Cur NPs groups had much larger necrosis regions compared with PEG-DOX NPs and DOX/Cur mixture groups, which demonstrated a higher anti-tumor activity, and this was consistent with the result of tumor growth inhibition.

## Discussion

We have developed a biocompatible prodrug nanoparticle for co-delivery of DOX and Cur. Various techniques including DLS, TEM, have been employed to verify successful fabrication of the prodrug nanoparticles. The drug loading content of DOX and Cur in the prodrug nanoparticle was 18.35% and 18.2%, respectively. Compared with other nanocarrier based combination chemotherapy system^45^,^46^, this prodrug nanoparticle had significantly higher drug loading content. The PEG-DOX-Cur NPs could release the active form of DOX and Cur more efficiently in acidic environment at pH 5.0 than at pH 7.4, as a result of the acid-labile Schiff’s base linkage between PEG and DOX. *In vitro* cytotoxicity and endocytosis studies carried out using HepG 2 and Hela cells demonstrated that the PEG-DOX-Cur NPs could be readily taken up by tumor cells. Better anti-tumor activity was also shown in cells treated with the nanoparticles than free DOX/Cur mixture or single drugs. Enhanced antitumor activity of the PEG-DOX-Cur NPs was also demonstrated in preliminary study using xenograft mice with hepatic tumors. Overall, the prodrug nanoparticle system provides a promising approach for combination therapy in cancer treatment. Subsequent studies will focus on the optimization of the drug dose to obtain the most effective dose with fewer side effects during the treatment, and the application of this prodrug system to various tumors.

## Methods

### Materials

Methoxy-poly (ethylene glycol)-aldehyde with a PEG molecular weight of 2000 was purchased from JenKem Technology Co., Ltd. (Beijing, China). Doxorubicin hydrochloride (DOX·HCl) and curcumin (Cur) were purchased from Wuhan Hezhong Biochem Co., Ltd. (Wuhan, China). Triethylamine and 4′,6-diamidino-2-phenylindole (DAPI) were provided by Sigma-Aldrich (St. Louis MO, USA). Other chemical reagents were analytic grade and used without further purification. The lysosome tracker was received from Sigma-Aldrich Chemical Co. (USA). The H&E stain kit was purchased from dakewe Technology Co., Ltd. (Beijing, China).

### Cell culture and animals

HepG 2 cells (human liver hepatocellular carcinoma cell line) and HeLa cells (human henrietta lackes strain of cancer cell line) were purchased from FDCC (Shanghai, China). The cells were cultured in DMEM medium supplemented with 10% FBS at 37 °C in 5% CO_2_ atmosphere. Culture dishes and 96 well plates were obtained from Corning (New York, USA). The BALB/c nude mice (male, 6–8 weeks) bearing HepG2 tumor model were received from Vital River Laboratory Animal Technology Co., Ltd. (Beijing, China). Mice were acclimated at 25 °C and 55% of humidity under natural light/dark conditions for 7 days before experiment. All the animal experiments were performed in accordance with the protocol approved by Chinese Academy of Medical Science and Peking Union Medical College, and adhered to the Guiding Principles in the Care and Use of Animals of the American Physiological Society.

### Synthesis and characterization of PEG-DOX

A typical Schiff’s base reaction was implemented to prepare the PEG-DOX. Briefly, methoxy-poly (ethylene glycol)-aldehyde (200 mg), deprotonated DOX (65 mg) and 21 μL of triethylamine were co-dissolved in DMSO (2.0 mL). The mixture was maintained for 24 h at 40 °C under magnetic stirring at 300 rpm, then the mixture was dialyzed (MWCO: 3.5 KDa) against DMSO to remove unreacted DOX and further dialyzed against PBS to remove DMSO. The PEG-DOX, a deep red powder, was obtained after lyophilization. The structure of PEG-DOX was characterized by ^1^H NMR (Varian INOVA).

### Preparation and characterization of PEG-DOX NPs and PEG-DOX-Cur NPs

PEG-DOX powders (30 mg) were dissolved in DMSO (5 mL) at 25 °C, and then the solution was dropwise added to 10 mL of PBS under gentle stirring. The solutions were dialyzed against excess PBS with a dialysis bag (MWCO: 1.0 KDa) for 48 h, and then filtered through a 450 μm pore-sized microporous membrane. The co-delivery of Cur was performed in a similar procedure with the co-dissolution of Cur (6 mg) in DMSO. The hydrodynamic size and morphology of obtained nanoparticles at a concentration of 2.0 mg/mL were characterized by DLS (Malvern Zetasizer Nano ZS) and TEM (JEM-2100F), respectively. The drug loading content in PEG-DOX-Cur NPs was determined by VARIOSKAN FLASH microplate reader (THERMO SCIENTIFIC) at 488 nm and 425 nm for DOX and Cur, respectively.

### Determination of pH-sensitive drug release

The drugs-release kinetics research of DOX and Cur from PEG-DOX-Cur NPs was monitored in PBS including 0.5% (w/w) Tween 80 at three different pH values (7.4, 6.5 and 5.0) by dialysis method. 5.0 mL of PEG-DOX-Cur NPs in different pH buffers was placed into dialysis bag (MWCO: 3.5 KDa) and was dialyzed against 40 mL of corresponding pH values buffers under gentle stirring at 37 °C. At predetermined time intervals, 5.0 mL of released solutions was taken out for testing and replenished with equivalent fresh buffers. The amount of DOX and Cur released from PEG-DOX-Cur NPs were detected by UV-visible spectrophotometry at 480 nm and 425 nm, respectively. Each sample in the release kinetics study was conducted in triplicate.

### Intracellular DOX and Cur release from PEG-DOX-Cur NPs

The cellular uptake behavior of PEG-DOX-Cur NPs was investigated after incubation for 2, 4 and 8 h. HepG 2 cells were placed into 24-well plates with a density of 10^4^ cells per well. After cultured for 24 h, the cells in each well were treated with PEG-DOX-Cur NPs. Then cell was washed with PBS (pH 7.4 10 mM) and fixed with paraformaldehyde for 20 min at 25 °C. The cell nucleus were counterstained by 4,6-diamidino-2-phenylindole (DAPI) with an excitation 405 nm according to standard procedure before imaged on fluorescence microscopy, DOX and Cur in different wells were observed at excitation of 540 nm and 505 nm, respectively. Meanwhile, in order to illuminate the mechanism of cellular uptake of the prodrug nanoparticle (PEG-DOX-Cur NPs), the colocalization experiment using lysosome staining reagent was carried out. The lysosome was stained with lysosome tracker after incubation for 0.5 h with PEG-DOX-Cur NPs, the image of lysosome tracker signal was visualized using a fluorescence microscopy with an excitation 577 nm.

For flow cytometric analyses, HepG 2 cells were seeded onto 24-well plate with a density of 10^5^ cells/well and then cultured in 5% CO_2_ atmosphere at 37 °C. After 24 h incubation, culture medium was replaced and cultured with free DOX, free Cur and PEG-DOX-Cur NPs at DOX concentration of 25.0 μg/mL and Cur concentration of 10.0 μg/mL, respectively. Cells incubated with PBS were used as blank control. Cells in new medium were incubated for 2, 4, 8 h at 37 °C, after washing three times with cold PBS cells were harvested by trypsin treatment at the indicated time points. The analysis was detected using flow cytometer on a FACS calibur (SD Biosciences US).

### *In vitro* anti-tumor efficiency assay

The *in vitro* cytotoxicity of PEG-DOX NPs, PEG-DOX-Cur NPs, free DOX and free Cur towards HepG 2 and HeLa cells were evaluated by MTT assay. Cells were seeded in 96-well plates at a density of 6000 cells per well in 0.1 mL DMEM solution and incubated in 5% CO_2_ atmosphere at 37 °C for 24 h, followed by removing culture medium and then adding 0.1 mL of medium containing different concentrations of drugs, such as PEG-DOX NPs, PEG-DOX-Cur NPs, free DOX, free Cur and free DOX/Cur mixture, cells incubated with 0.1 mL of PBS were used as control. After 24 h incubation, the medium were discarded and 20 μL of MTT solution was added to the cells for another 4 h, the absorbency of the medium solution were measured on a microplate reader at 570 nm. The cell viability was expressed by (sample/control) × 100%. All samples are presented as average ± SD (n = 6).

### *In vivo* drug accumulation and penetration studies

When the tumor volume of the BALB/c nude mice reached 500 mm^3^, they were injected with PEG-DOX-Cur, free DOX and free Cur via tail vein at equivalent DOX dose of 5 mg/kg and at equivalent Cur dose of 2 mg/kg, respectively. At indicated time points (1 h, 8 h, 24 h) mice were sacrificed, the tumor and major organs (heart, liver, spleen, lung and kidney) were harvested. The fluorescence intensity of DOX in excised organs and tumor were examined using Kodak IS *in vivo* imaging system. The concentration of Cur in tumors were measured by LC-MS/MS according to previous report^47^.

After 24 h post injection, we harvested tumor and fixed it with 4% paraformaldehyde for 24 h at 4 °C. In order to study drug accumulation and penetration through tumor sections, tumor was embedded in paraffin, cut and mounted 3–5 μm thick sections onto microscopic slides, the sections were stained with DAPI at 20 μg/ml for 15 min. DOX in tumor tissues was observed by optical microscope. Four random fields were selected for statistical analysis using Image J version 1.42 (NIH Bethesda, MD), which the low intensity fluorescence was used as the background, and high intensity spots as the fluorescence signals of DOX^48^.

### *In vivo* anti-tumor efficiency

The BALB/c nude mice (male, 6–8 weeks) bearing HepG2 xenografts were randomly divided into four groups (n = 10), which was PBS, DOX/Cur mixture (5 mg DOX/kg and 2 mg Cur/kg), PEG-DOX NPs (5 mg DOX/kg), PEG-DOX-Cur NPs (5 mg DOX/kg and 2 mg Cur/kg), respectively. Mice in different treatment groups were intravenously via tail vein with different formulations at days 0, 4, 8 and 12. In order to evaluate the anti-tumor activities of different treatment groups, the length and width of tumor and body weight of mice were measured every other day. Tumor volume (V) was calculated using the formula: V (mm^3^) = 1/2 × length (mm) × width (mm)^2^. After 18 days measurement, mice were sacrificed, tumors were dissected and fixed in 4% paraformaldehyde for 24 h. Samples were dehydrated through a graded series of ethanol and embedded in paraffin. The samples were cut into 8 μm thick sections and stained with H&E for histological analyses. The photos were taken by an optical microscope (Leica DMI6000 B, Germany).

### Statistical analysis

One way analysis of variance was used for the statistical analysis. *p < 0.05 and **p < 0.01 were utilized for statistical significance. All data were shown as mean ± standard deviation values.

## Additional Information

**How to cite this article**: Zhang, Y. *et al.* Co-delivery of doxorubicin and curcumin by pH-sensitive prodrug nanoparticle for combination therapy of cancer. *Sci. Rep.* **6**, 21225; doi: 10.1038/srep21225 (2016).

## Figures and Tables

**Figure 1:**
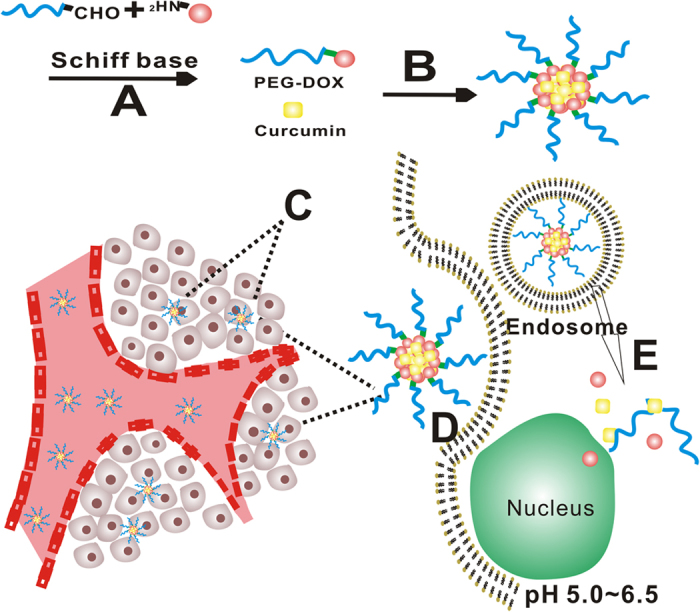
Schematic illustration of the synthesis and working principle of PEG-DOX-Cur NPs. (**A**) Synthesis of PEG-DOX NPs *via* Schiff’s base. (**B**) Preparation of PEG-DOX-Cur NPs by nanopreciptated technique. (**C**) Passive tumor targeting was achieved by EPR effect. (**D**) The PEG-DOX-Cur NPs could be internalized by cancer cells through endocytosis. (**E**) DOX and Cur were released with the cleavage of the Schiff’s base in tumor cells and diffused into nucleus.

**Table 1:** Chemical character of PEG-DOX and PEG-DOX-Cur NPs.

Sample	DLC of DOX ^a^	DLC of DOX ^b^	DLC of Cur ^b^	DLE of Cur ^c^	Size ^d^ (nm)	PDI ^e^	ζ ^f^ (mV)
PEG-DOX NPs	20%	18.72%	-	-	161.8 ± 2.5	0.07 ± 0.01	−.34 ± 0.03
PEG-DOX-Cur NPs	20%	18.35%	18.2%	91%	183.5 ± 4.5	0.14 ± 0.04	−.68 ± 0.06

**Table 2:** The IC_50_ values of different agents toward HepG 2 cells and Hela cells as measured with MTT assay.

Sample	HepG 2 cells		Hela cells	
	IC_50_DOX/(μg/mL)	IC_50_Cur/(μg/mL)	IC_50_DOX/(μg/mL)	IC_50_Cur/(μg/mL)
Cur	-	0.853	-	0.839
DOX	2.112	-	2.218	-
PEG-DOX NPs	2.530	-	2.851	-
DOX/Cur mixture	2.146	0.845	2.257	0.887
PEG-DOX-Cur NPs	1.700	0.680	1.741	0.697
